# Real-time visual intelligence for defect detection in pharmaceutical packaging

**DOI:** 10.1038/s41598-024-69701-z

**Published:** 2024-08-13

**Authors:** Ajantha Vijayakumar, Subramaniyaswamy Vairavasundaram, Joseph Abraham Sundar Koilraj, Muthaiah Rajappa, Ketan Kotecha, Ambarish Kulkarni

**Affiliations:** 1 School of Computing, SASTRA Deemed University, Thanjavur, 613401 India; 2 School of Computer Science and Engineering, Vellore Institute of Technology, Vellore, 632014 India; 3 Symbiosis Centre for Applied Artificial Intelligence, Symbiosis Institute of Technology, Symbiosis International University, Pune, 411045 India; 4 School of Engineering, Swinburne University of Technology, Hawthorn, Australia

**Keywords:** YOLOv8, Object detection, Computer vision, Defect detection, Coordinate attention, Computational models, Data acquisition, Data integration, Health services, Quality of life

## Abstract

Defect detection in pharmaceutical blister packages is the most challenging task to get an accurate result in detecting defects that arise in tablets while manufacturing. Conventional defect detection methods include human intervention to check the quality of tablets within the blister packages, which is inefficient, time-consuming, and increases labor costs. To mitigate this issue, the YOLO family is primarily used in many industries for real-time defect detection in continuous production. To enhance the feature extraction capability and reduce the computational overhead in a real-time environment, the CBS-YOLOv8 is proposed by enhancing the YOLOv8 model. In the proposed CBS-YOLOv8, coordinate attention is introduced to improve the feature extraction capability by capturing the spatial and cross-channel information and also maintaining the long-range dependencies. The BiFPN (weighted bi-directional feature pyramid network) is also introduced in YOLOv8 to enhance the feature fusion at each convolution layer to avoid more precise information loss. The model's efficiency is enhanced through the implementation of SimSPPF (simple spatial pyramid pooling fast), which reduces computational demands and model complexity, resulting in improved speed. A custom dataset containing defective tablet images is used to train the proposed model. The performance of the CBS-YOLOv8 model is then evaluated by comparing it with various other models. Experimental results on the custom dataset reveal that the CBS-YOLOv8 model achieves a mAP of 97.4% and an inference speed of 79.25 FPS, outperforming other models. The proposed model is also evaluated on SESOVERA-ST saline bottle fill level monitoring dataset achieved the mAP50 of 99.3%. This demonstrates that CBS-YOLOv8 provides an optimized inspection process, enabling prompt detection and correction of defects, thus bolstering quality assurance practices in manufacturing settings.

## Introduction

The pharmaceutical industry employs new techniques in manufacturing processes to develop quality products. Consequently, decreasing the risk of developing faulty products. The sector is transitioning from Pharma 3.0 to Pharma 4.0, marking a significant shift in quality management approaches^1^. While Pharma 3.0 relied on semi-automated processes with some human involvement, it had limitations in defect detection due to factors like human error, inconsistent lighting, tablet size variations, and fatigue. This is a critical issue, as identifying faulty tablets before packaging is essential. Pharma 4.0 addresses these challenges by incorporating high levels of automation and data analysis, powered by artificial intelligence (AI) and the Internet of Things (IoT). AI algorithms can process sensor data in real-time, enabling faster and more accurate defect identification compared to human inspectors. Computer vision (CV) technology further enhances this capability by training AI systems to replicate human vision using live video streams. This technological advancement overcomes traditional defect detection issues, leading to improved quality, increased production efficiency, enhanced safety, reduced operational costs, and decreased reliance on manual labor^2^. The impact of these innovations is reflected in the projected growth of the pharmaceutical packaging market, which is expected to reach $229.9 billion by 2027, up from $99.9 billion in 2021. This substantial growth is likely driven by the increasing adoption of Pharma 4.0 technologies, which ensure consistent quality and minimize waste in the production process.

YOLO (you only look once) has emerged as a pivotal network model for object detection, particularly excelling in real-time applications due to its impressive accuracy and speed^3^. Object detection has gained significant progress in the field of computer vision (CV), which offers reliable means of locating and identifying objects in both images and videos^4^^,^^5^. YOLO is designed to provide object coordinates within an image it has been trained on, along with a confidence score indicating the prediction's reliability. Its applications span various domains, including defect detection in surveillance, autonomous driving, manufacturing, and augmented reality, highlighting its pivotal role in numerous contexts. In manufacturing, defect detection is vital for maintaining product quality, reducing waste, and protecting consumers by identifying flaws throughout the production process. Object detection algorithms can be categorized into two distinct categories based on how frequently they analyze an input image: single-stage detectors and two-stage detectors^6^. Single-stage detectors aim to identify objects across all possible spatial areas in a single step, using a simpler architecture for performing object detection. Conversely, two-stage detectors utilize more complex architecture with dual passes to suggest approaches for identifying precise regions. The YOLO family has evolved through multiple iterations since its initiation, with each subsequent new version building upon the predecessors to tackle detection challenges and enhance overall performance.

In the realm of manufacturing, tablets serve as indispensable remedies for human sicknesses. As disease prevalence increases, so does the demand for these pharmaceutical products. To meet this growing need effectively, continuous manufacturing processes for tablets are imperative. However, this production process is not without its challenges, as defects can compromise the quality of the tablets. Traditionally, the detection of defects in tablets during the manufacturing process has depended on human inspection. However, this method is susceptible to inaccuracies. To mitigate this problem, computer vision (CV) technologies have been implemented, providing automated inspection capabilities. From these, the YOLO network model is best option for its ability to detect defects in real-times scenario. YOLOv8, a variant of the YOLO algorithm, is considered predominantly effective in this esteem due to its enhanced accuracy and suitability for real-time applications^7^. Its adoption is expected to streamline the inspection process, ensuring defects are identified and corrected promptly, thereby improving quality assurance protocols within the manufacturing industry.

The proposed CBS-YOLOv8 model aims to improve the accuracy and speed of detecting tablet defects in pharmaceutical blister packages. The model's effectiveness is evaluated by training and testing it on a custom dataset created with defective tablets within blister packages and used the SESOVERA-ST saline bottle fill level monitoring dataset to analyse and obtain the effectiveness of the proposed model. To achieve an optimal real-time object detector, the CBS-YOLOv8 model incorporates the following contributions:

1. To enhance the feature extraction capabilities of the YOLOv8 model for detecting defects in pharmaceutical tablets, the coordinate attention mechanism was integrated.
2. A BiFPN is added to the backbone of the YOLOv8 model to improve the feature fusion in diverse scales.
3. Finally, the SPPF in the backbone of YOLOv8 is replaced with SimSPPF to reduce the computational complexity and make the model computation faster.

The remainder of the document is structured as follows: The second section summarizes related research work. The third section describes the architecture of YOLOv8. The proposed CBS-YOLOv8 model is analyzed with its architecture, and the overall process done in the proposed model is described in section four. The fifth section describes the dataset of blister packages used in this model. Experiments (comparison and ablation) are carried out in the dataset, and the analysis is done based on experimental results. Finally, the conclusion of the research work is given in the sixth section.

## Related work

In the past years, defect detection has been done based on machine learning, and deep learning is used widely to find defects in various objects, such as pavement crack detection, vehicle detection, steel surface detection, and tablet coating defect detection. Hsiung-Cheng Lin et al.^8^ proposed a defect identification model in tablets using a technique called biaxial planes discrete scanning model. The defect on the tablet is identified using SME (similarity gap and square mean error) in between the intact tablet and the defective tablet. Domen Racki et al.^9^ developed a TriNet architecture that has a CNN model for segmentation and surface detection by using different receptive fields, achieving a real-time environment by reducing computational complexity. Tipu Sultan et al.^10^ introduced ultrasonic reflection ray tracing to monitor the tablet quality in real-time. They also developed varying axial load–displacement measurements for monitoring the design and development of oral solid dosage forms. Sora Kim et al.^11^ proposed a pill and defect detection module for pill segmentation and defect identification in pills, respectively. They also proposed a patch division method to reconstruct the unseen data effectively because the model is trained only on normal data. Lilla Alexandra Meszaros et al.^12^ developed a machine vision system using UV/VIS images to identify the particle size variation in intact tablets. They developed a two image processing algorithm for UV and VIS images. For classification, a pattern recognition neural network is applied, which is based on particle size differences. Finally, they achieved a classification result for both VIS and UV images.

However, tablet defect detection within the blister packages using deep learning techniques is very rare in literature. Studies were made on image processing techniques to identify tablet defects, which give less accuracy and are not suitable for live stream videos because the pharmaceutical industry manufactures tablets continuously. Mate Ficzere et al.^13^ used the YOLOv5 model for identifying the coating defects in tablets, while the tablet diameter was identified using an image analysis technique to calculate the weight gain of coated tablets. Yonten Jamtsho et al.^14^ used the YOLOv2 model for the detection of the license plate of a motorcyclist who is not wearing a helmet in real-time. They also proposed a method for centroid tracking with a line to eliminate a false positive when the motorcyclist leaves the video frame. Krisha Bhambani et al.^15^ developed a YOLO detection modelto identify instances of face mask violations and social distancing violations depicted in videos or images. The above research focused on using the YOLO family for detecting the defects in tablets and not covering more of the defects that affect the visual appearance of tablets, which leads to false positives or false negatives. In terms of using the YOLO family, accuracy and inference speed are achieved.

Duo Ma et al.^16^ proposed a YOLO-MF, which is the improved version of YOLOv3to detect cracks in the pavement, and MF is used to track the cracks identified in the video to avoid the counting loop of cracks. They also developed a PCGAN to generate more realistic images of pavement cracks because of the difficulty in collecting real crack images. Yang wang et al.^17^ proposed an improved version of the YOLOv7 model to identify the defects in the steel surfaces and also focus on increasing the detection speed and detection accuracy of conventional defect detection in steel surfaces. The improvements in YOLOv7 include adding deweighted BiFPN to the neck part, ECA attention mechanism in the backbone, and SIoU loss function in the head part. These improvements increase the speed and accuracy of the detection of steel. Mohammad Hossein Hamzenejadi et al.^18^ proposed a real-time detection of vehicles in UAV images by performing the architecture modifications and performance boosting on YOLOv5. The detection accuracy is increased by adding an SE attention mechanism in the backbone and alpha IoU loss function in the head part. Then, the inference speed is increased by incorporating the ghost convolution and adjusting the width and height of the network. Taiguo Li et al.^19^ proposed an upgraded deep learning model by incorporating the SE attention mechanism and BiFPN within YOLOv5 architecture to find the driver distraction while driving.

However, research was done on different applications to improve the accuracy and speed of a model by enhancing the YOLO model. To effectively detect the defects and classify them with finer details in pharmaceutical blister packages and to maintain a good trade-off between the speed in detection and accuracy while detection, need to develop a detection model with low computation, fewer parameters, and high inference speed. Therefore, this research work develops the CBS-YOLOv8 model, designed for real-time detection of defects in tablets within blister packages.

## Fundamental architecture of YOLOv8

**Figure 1:**
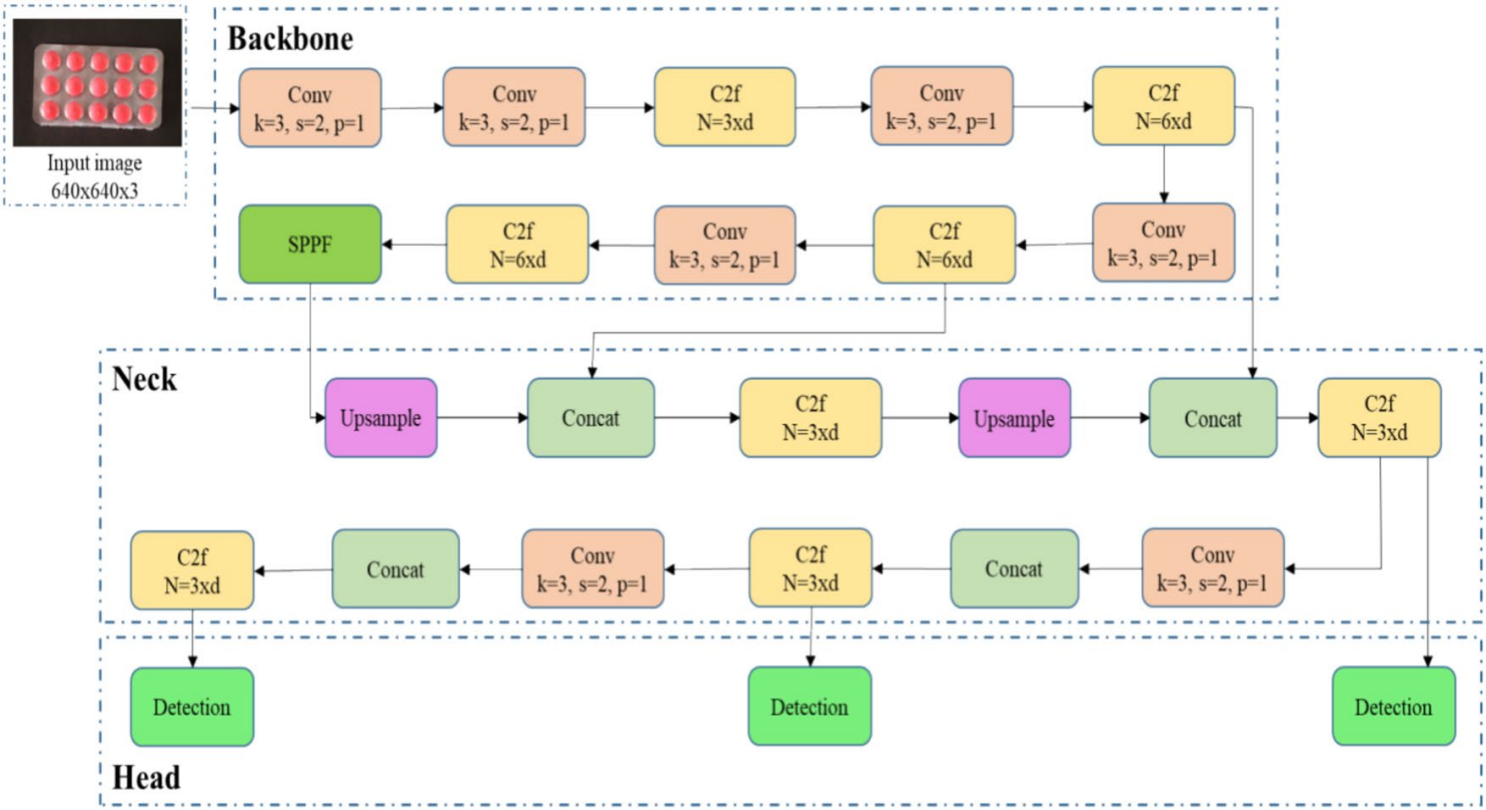
Architecture of YOLOv8. Inspired from^24^.

**Figure 2:**
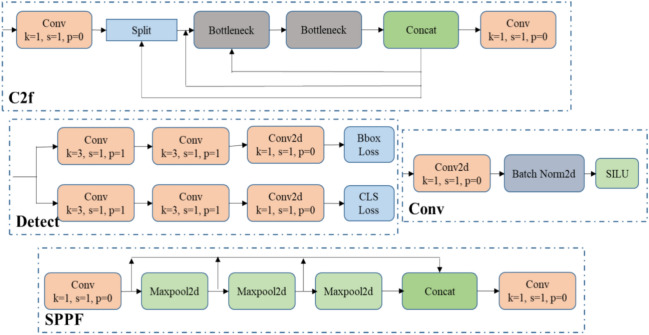
Each component details (YOLOv8). Inspired from^24^.

YOLOv10 is the latest object detection variant of the You Only Look Once (YOLO) family. YOLO faces several iterations, from YOLO to YOLOv8, then YOLO NAS, YOLOv9 and recently YOLOv10 have been introduced. However, YOLOv8 is more suitable to the proposed application and chosen it for developing the enhanced defect detection model. Using a single neural network, the YOLOv8 architecture predicts the labels of objects and bounding boxes within an image. It has several vital components that make up the YOLOv8 architecture, such as the input part, backbone, neck, and head^7^ is shown in the Figs. 1 and 2.

- *Input part* The notable issue nowadays is the diverse image sizes (in terms of height and width) that are available in the dataset. To improve the image data processing efficiency, the YOLOv8 model preprocesses the input data to adapt their predefined scaling. The default input image size for the YOLOv8 model is set to 640 × 640. This module also includes functions that allow users to choose the most effective data enhancement approach for their dataset, such as mosaic data augmentation and adaptive anchor frame computation. YOLOv8 performs mosaic augmentation while training, where the four images are stitched and enforced to learn the new location and occlusion of the object. This mosaic augmentation stopped before 10 epochs while training because of performance degradation.
- *Backbone* The backbone is responsible for extracting the purposeful features from the input data. This network consists of three modules: Conv module, C2f module, and SPPF module. The Conv module includes the three functions- Conv2d, BN2d, and SILU activation function. YOLOv8 incorporates a C2f block that integrates existing functions like C3 and ELAN, optimizing gradient flow. The model also uses a spatial pyramid pooling fast (SPPF) module, which employs maximum pooling with different kernel sizes to extract multi-scale features. The structure, as shown in Fig. 2, comprises Conv, C2f, and SPPF components.
- *Neck* The neck of YOLOv8 employs a fusion approach that integrates the PAN (path aggregation network) and the FPN (feature pyramid network) to efficiently merge features across different scales. This combination facilitates the upsampling and downsampling processes to blend the features. The FPN integrates a horizontal connection on top of the backbone to establish a pathway for upscaling features, merging features from lower levels with those from higher levels. This design enables the network model to detect objects across various scales while preserving semantic details. Conversely, the PAN structure aims to emphasize precise localization conveyed by low-level features, then it is combined to the rich semantic information of FPN. The PAN accomplishes this by merging features of varying resolutions through both horizontal and vertical pathways, facilitating the effective transfer of low-level features to higher levels and preserving positional information. For implementation purposes, the PAN structure employs two 3 × 3 standard convolutions and three C2F modules to transform features, reducing the feature map size from 80 × 80 to 20 × 20 and concatenating features from the FPN.
- *Head* The YOLOv8’s head section comprises three detection blocks, each composed of two divisions. Within each division, there are two CBS layers, a 2D convolution layer, and losses for classification and bounding box. These detection modules implement an anchor-free, dynamic Task Aligned Assigner. Once the neck section produces three feature maps of varying image sizes, each of these maps is fed into one of the three detection modules. From there, direct regression for classification and bounding boxes is executed on the objects within the detection module^20^.
- *Loss function* The head part of YOLOv8 includes two loss functions- classification loss and regression loss. The classification loss has a binary cross entropy (BCE), which measures the discrepancy between the ground truth labels and the predicted class probabilities. It helps the model accurately classify the detected objects. The regression loss employs a distribution focal loss (DFL)^21^ along with a complete intersection over union (CIOU) loss function. CIOU evaluated the difference between the ground truth and predicted bounding boxes, considering not just the overlap area but also the aspect ratio and the distance between the box centers. This enhances the model's capacity for accurately indicative object locations. DFL solves the issues that arise from the class imbalance problem^22^ and also increases the bounding box regression even for blurry boundaries if they are difficult to predict. DFL estimates the probability of boundaries of bounding boxes rather than fixed-sized bounding boxes, and it specifies if there are any uncertainties in positions. YOLOv8 utilizes a task-aligned assigner^23^ to calculate a metric for aligning tasks, integrating regression coordinates and classification scores into the calculation. These metrics merge classification scores and Intersection over Union (IoU) value, allowing concurrent enhancement of both localization and classification, although reducing the effect of lower quality detection boxes. In object detection, IoU is a commonly used performance evaluation metric employed to determine the distance between the ground truth and the predicted bounding boxes. If the IoU value exceeds 0.5, then the object is classified as detected. The IoU is expressed as,
  1 $$IoU=\frac{|A\cup B|}{|A\cap B|}.$$

## CBS-YOLOv8 defect detection model

The proposed CB-YOLOv8 detection model integrates a coordinate attention mechanism, BiFPN and SimSPPF. Incorporating the coordinate attention mechanism within the YOLOv8 backbone enhances object localization by prioritizing relevant spatial coordinates, thereby yielding refined feature maps. The inclusion of BiFPN in the YOLOv8 neck facilitates efficient feature aggregation across multiple scales, leading to improved multi-scale prediction capabilities. SimSPPF reduces the computational cost. Because the usage of the CA mechanism will increase the computational complexity by adding it to the backbone of YOLOv8.

### Coordinate attention mechanism

The idea of attention draws inspiration from the visual attention system found in humans, which extracts the most relevant information from huge amounts of data. The effective features are extracted by highlighting the most important information and reducing the less relevant data. Incorporating the attention in the appropriate location of the backbone can reduce the impact of distracting background data, increase the accuracy of targeted feature retrieval, and ultimately raise the precision of the algorithm's detection capabilities. Various attention mechanisms are available nowadays, such as SE attention^25^, ECA attention^26^, and CBAM attention^27^. SE (squeeze and excitation) attention, which calculates the channel attention using 2D global pooling, was resulting in significant performance improvements with minimal computational requirements. The SE attention mechanism focuses only on encoding inter-channel data. Still, it lacks focus on positional information, which leads to critical in capturing the structure of objects in computer vision tasks. ECA, or Efficient Channel Attention, enhances SE attention through the utilization of one-dimensional convolutional layers for gathering cross-channel data, resulting in more precise attention details. However, ECA neglects the positional data of image characteristics, which limits its effectiveness. The CBAM (Convolutional Block Attention Module), employing a convolutional block attention mechanism, integrates both channel and spatial aspects, strengthening the correlation between channel features and spatial dimensions. Nevertheless, it struggles to capture the contextual information surrounding the target. Additionally, the compact attention models focusing solely on the channel domain mentioned earlier solely address individual channel data, disregarding the positional information within the image. Although CBAM incorporates both channel and positional details, it cannot capture long-range relationships^28^.

**Figure 3:**
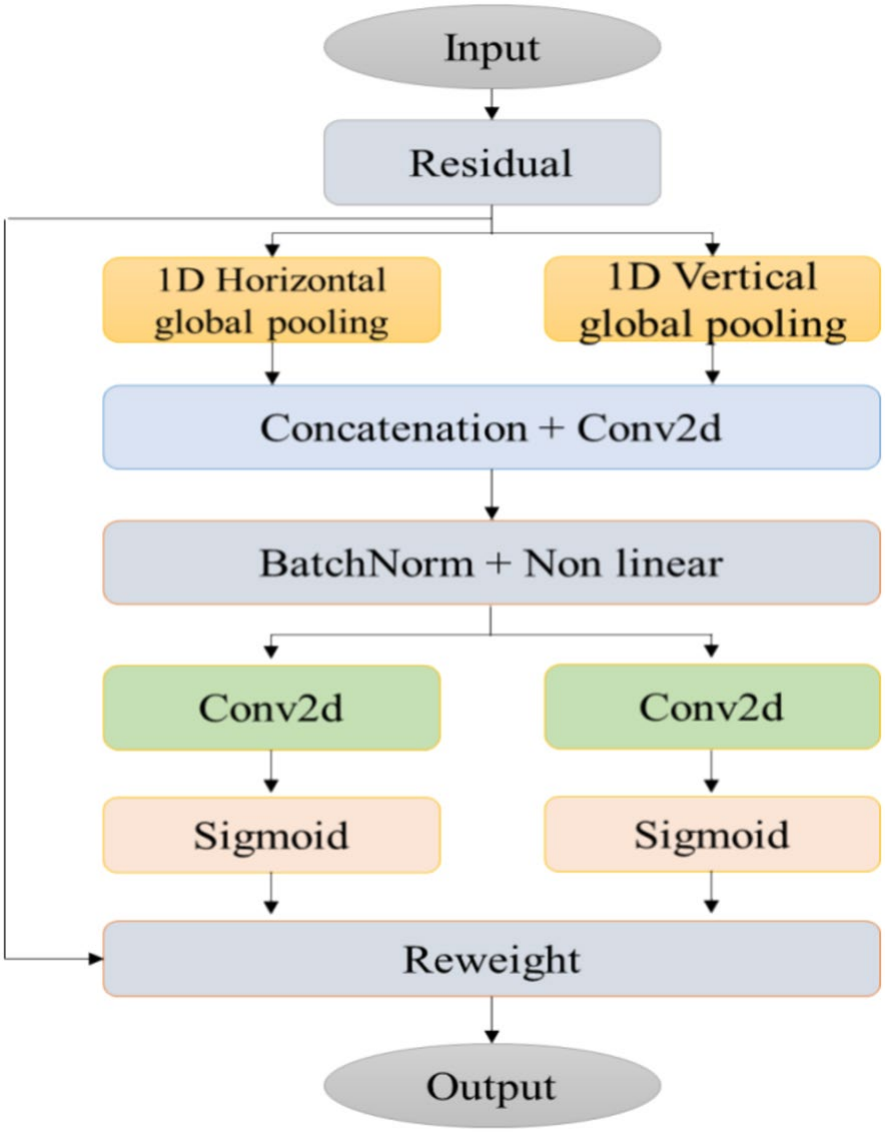
Structure of coordinate attention mechanism.

Coordinate attention (CA) is a fast, simple, lightweight mechanism that is easily adaptable to integrate with the core structure of any algorithm^29^. It balances both long-range positional relationships and channel information and engages with broader contexts without significant computational overhead. This enhancement leads to improved target detection and recognition, surpassing other attention mechanisms like SE, ECA, and CBAM. Insert the Coordinate Attention module after the convolutional layers within the C2f block, this enables the network model to concentrate on appropriate features at various stages of feature extraction. The process separates the attention mechanism into two unique one-dimensional feature encoding operations, each dedicated to collecting features across different spatial dimensions. This approach successfully creates an attention map that is aware of coordinates by identifying operational coordinates, which helps in capturing spatial dependencies over long distances. The resulting feature maps are then converted into attention maps that are conscious of both the direction and the location, thus enhancing the depiction of target objects without adding to the computational load. Through dividing channel attention into two parallel one-dimensional features, this coordinated attention method highlights the reduction of positional information that occurs due to global pooling. The whole structure of CA mechanism is shown in the Fig. 3. The CA mechanism includes two steps: coordinate information embedding and coordinate attention generation.

#### Coordinate information embedding

In channel attention mechanisms, global pooling is a common technique utilized to integrate spatial information spanning the entirety of an image. However, it compresses this comprehensive spatial data to a channel descriptor, making it challenging to maintain precise positional information crucial for capturing spatial data in the visualization process. To promote the attention blocks' ability to grasp long-range spatial relations while preserving accurate positional details, the CA module factorizes the global pooling represented in SE attention into two separate one-dimensional feature encoding processes. Specifically, the X is the provided input, which is fed into two spatial pooling kernels (H, 1) and (1, W) are used for the encoding process of vertical and horizontal coordinates, respectively. Then, the cth channel output for H and W can be formulated as

2 $${z}_{c}^{h}\left(h\right)= \frac{1}{W}\sum_{0\le i<w}{x}_{c}\left(h,j\right),$$

3 $${z}_{c}^{w}\left(w\right)= \frac{1}{H}\sum_{0\le j<H}{x}_{c}(j,w).$$

The above two Eqs. (1) and (2) aggregate features in two directions individually, yielding a set of direction aware feature maps. The above two equations make this attention to capture positional information in one spatial direction and long-range information in another spatial direction, which helps the model to accurately locate the interested objects in an image or video.

#### Coordinate attention generation

By using the aggregated features from the Eqs. (1) and (2), first perform the concatenate process and send that feature maps to individual 1 × 1 convolution function F1, gives

4 $${\varvec{f}}= \delta \left(F1\left(\left[{{\varvec{z}}}^{h}, {{\varvec{z}}}^{w}\right]\right)\right), {\varvec{f}}\epsilon {\mathbb{R}}^{C/r\times (H+W)},$$

where $\delta (.)$ is a nonlinear activation function, **z** represents the concatenation process, and r is the reduction rate used to control the block size. Then split the **f** into two individual tensors such as horizontal and vertical coordinates ${{\varvec{f}}}^{{\varvec{h}}}\epsilon {\mathbb{R}}^{C/r\times H}$ and ${{\varvec{f}}}^{{\varvec{w}}}\epsilon {\mathbb{R}}^{C/r\times w}$ respectively. Again, additional separate two 1 × 1 convolution operations F_h_ and F_w_ are used to transmute separately ${{\varvec{f}}}^{{\varvec{h}}}$ and ${{\varvec{f}}}^{{\varvec{w}}}$ to a similar channel number to the X input, gives

$${g}^{h}= \sigma \left({F}_{h}\left({{\varvec{f}}}^{{\varvec{h}}}\right)\right),$$

5 $${g}^{w}= \sigma \left({F}_{w}\left({{\varvec{f}}}^{{\varvec{w}}}\right)\right),$$

where σ is the sigmoid function. To reduce the model complexity by decreasing the rate of reduction r (eg., r = 32). The output of the equations ${g}^{h}$ and ${g}^{w}$ are extended and employed as an attention weight. At last, result of CA module Y is calculated by using ${g}^{h}$ and ${g}^{w}$ and it is written as

6 $${y}_{c}\left(i,j\right)= {x}_{c}\left(i,j\right)\times {g}_{c}^{h}\left(i\right)\times {g}_{c}^{w}\left(j\right).$$

### Bi-directional feature pyramid network

**Figure 4:**
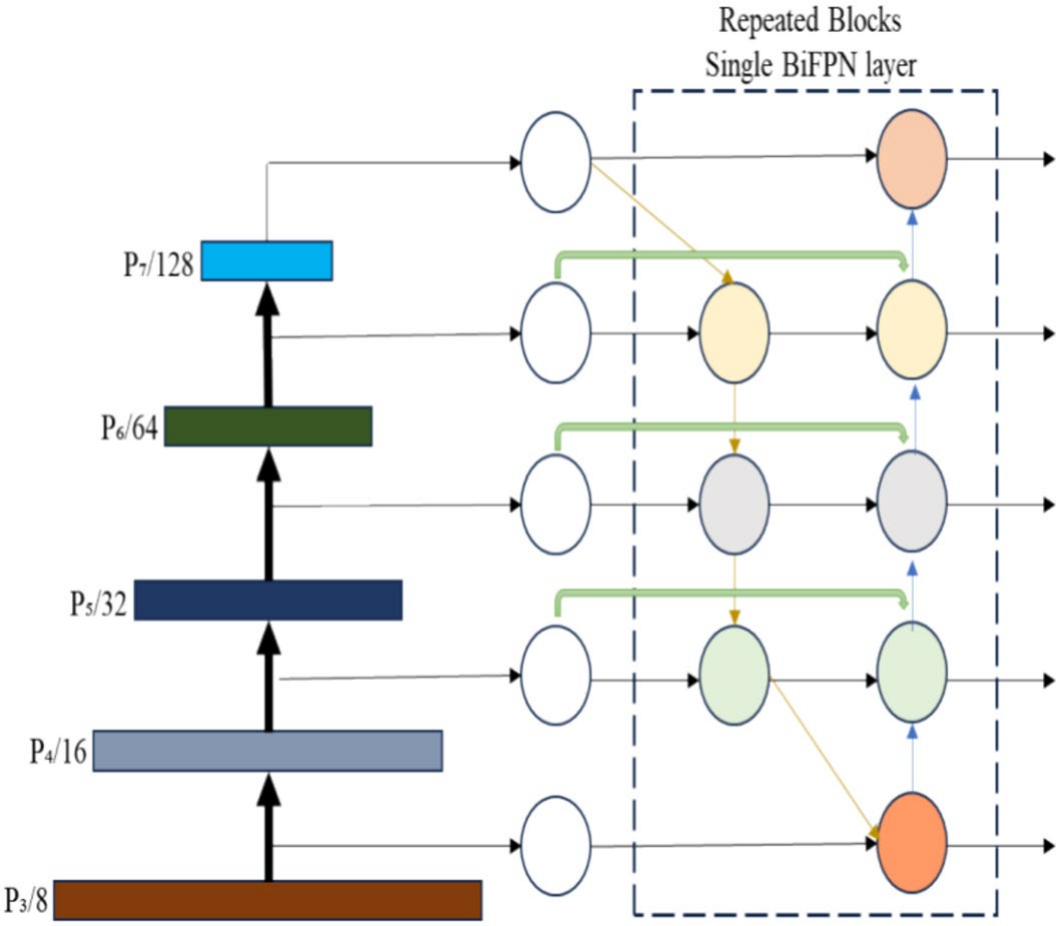
BiFPN layer structure.

Feature pyramid network (FPN) is mainly utilized for different scale feature fusion. NAS- FPN and PANet are also developed for feature fusion used to obtain a cross scale feature fusion. Previous research has typically combined various input features by simply adding them together without distinct characteristics. However, given that these features are captured at different resolutions, the notable issue is that they often contribute unequally to the final combined feature. To tackle this challenge, a straightforward yet remarkably efficient solution is called the weighted bi-directional feature pyramid network. This approach incorporates trainable weights to understand the significance of individual input features, while iteratively merging features across multiple scales in both bottom-up and top-down directions. BiFPN is a feature fusion method that is introduced in EfficientDet-D7 which is an object detector that achieves 55.1 AP on the COCO dataset^30^. Its structure is shown in the Fig. 4. The BiFPN includes two steps: efficient bidirectional cross scale connection and weighted feature fusion.

#### Efficient bidirectional cross scale connection

FPN uses only a top-down approach for feature fusion. Lower-level features have higher and better resolution but less semantic data (good for localization). Higher-level features have robust semantic information (good for recognition) but lesser resolution. To solve this problem, PANet includes a bottom-up approach after the top-down approach that is mentioned in FPN. Cross-scale connections give better feature fusion. So, NAS-FPN designs a new neural architecture for achieving cross-scale feature fusion. Still, its architecture is complex and difficult to modify and needs a greater number of GPU hours for searching. To the core, PANet gives better results than NAS-FPN and FPN, but the lacking of PAN requires more computations and parameters. To solve this issue, BiFPN is developed which includes three major changes. First, if a node has a single input edge without feature fusion, then that node is removed. Second, if the node (both actual input and output) is at the same level, then an additional edge is added between these nodes, which enhance the feature fusion. Third, the BiFPN layer is repeated multiple times to achieve the high-end feature fusion. The number of times needed to repeat the BiFPN layer is based on resource constraints by using a compound scaling technique.

#### Feature fusion with weighted value

The diverse input features with different resolutions lead to inequality in output features. So, the BiFPN adds the weighted value to each input feature and strengthens the model by understanding the importance of every input feature. In BiFPN, three weighted values are used for fusion:

- *Unbounded fusion* is represented as $O= \sum_{i}{w}_{i} . {I}_{i}$*,* where w_i_ can be a vector, multi-dimensional tensor, scalar that represents a learnable weight for per channel, pixel, feature. A constraint arises when weights are represented as scalar values, potentially causing instability during training. However, employing weight normalization offers a viable strategy to limit the weight value ranges and overcome this issue.
- *Softmax based fusion* is represented as $O= \sum_{i}\frac{{e}^{{w}_{i}}}{\sum_{j}{e}^{{w}_{j}}} . {I}_{i}$*,* Softmax function is applied to all weights, so it normalizes the weights from 0 to 1.
- *Fast normalized fusion* is represented as $= \sum_{i}\frac{{w}_{i}}{\in + \sum_{j}{w}_{j}} . {I}_{i}$, the ReLU is applied after each weight w_i_ which ensures w_i_ ≥ 0 and ϵ is set to 0.0001 is small value for avoiding the numerical instability.

Therefore, the final BiFPN combines the above two steps. For example, the feature fusion of layer 6 for BiFPN is represented as

7 $${P}_{6}^{td}=Conv\left(\frac{{w}_{1} . {P}_{6}^{in}+ {w}_{2} . Resize({P}_{7}^{in}}{{w}_{1}+ {w}_{2}+ \in }\right),$$

8 $${P}_{6}^{out}=Conv\left(\frac{{w{\prime}}_{1} . {P}_{6}^{in}+ {w{\prime}}_{2} . {P}_{6}^{td}+ {w{\prime}}_{3} . Resize\left({P}_{5}^{out}\right)}{{w{\prime}}_{1}+ {w{\prime}}_{2}+ {w{\prime}}_{3}+ \in }\right).$$

${P}_{6}^{td}$ is a layer 6 intermediate feature in top down approach and ${P}_{6}^{out}$ is a layer 6 output feature in bottom up approach.

### SimSPPF loss function

**Figure 5:**
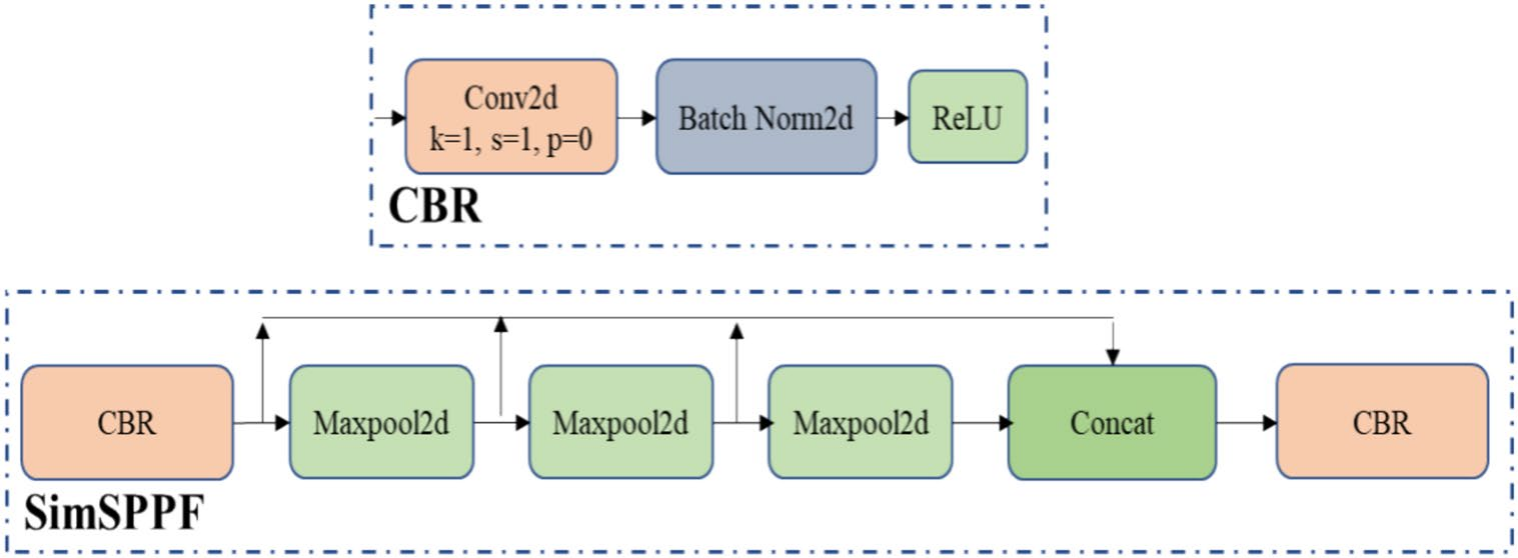
Structure of SimSPPF.

To ensure the real-time performance of the tablet defect detection, the SPPF in the YOLOv8 backbone is replaced with SimSPPF. In YOLOv6, the SimSPPF is first introduced is shown in the Fig. 5, which is an enhanced version of SPPF, it reduces the computational complexity and inference time^31^. This will be achieved by aggregating three 5 × 5 maximum pooling layers to process each input, gives a finite-sized feature map. These feature maps enhance the feature representation and the receptive field of the model. The conv module in YOLOv8’s SPPF includes a convolution layer, batch normalization, and Sigmoid Linear Unit (SiLU) activation function (CBS module). But the SimSPPF has a conv module which includes a convolution layer, batch normalization and Rectified Linear Unit (ReLU) activation function (CBR module). The equation for SiLU and ReLU activation functions are,

9 $$SiLU, f\left(x\right)= \frac{{x}^{2}}{1+{e}^{-x}},$$

10 $$ReLU, f\left(x\right)= \left\{\begin{array}{c}x, if x>0\\ 0, if x\le 0\end{array}=\text{max}(0,x)\right..$$

The computation of exponential terms in SiLU leads to high computational complexity. To mitigate this issue, replace the SiLU with the ReLU function in the conv module in order to increase the Frames Per Second (FPS).

### CBS-YOLOv8

Overall, the enhancements and refinements made earlier have been integrated into the architecture of the CBS-YOLOv8 network, resulting in notable enhancements in its performance and accuracy of the detection model. Incorporation of CA mechanism in shallow and before bottleneck layer of backbone enables more extraction of features. The BiFPN module expands the receptive field of the model by using high-resolution features. This will increase the detection of tiny cracks in tablets within the blister packages. The SimSPPF reduce the computation complexity which ensures the real-time object detection in the pharmaceutical industry, leading to a more accurate and robust detection system.

**Figure 6:**
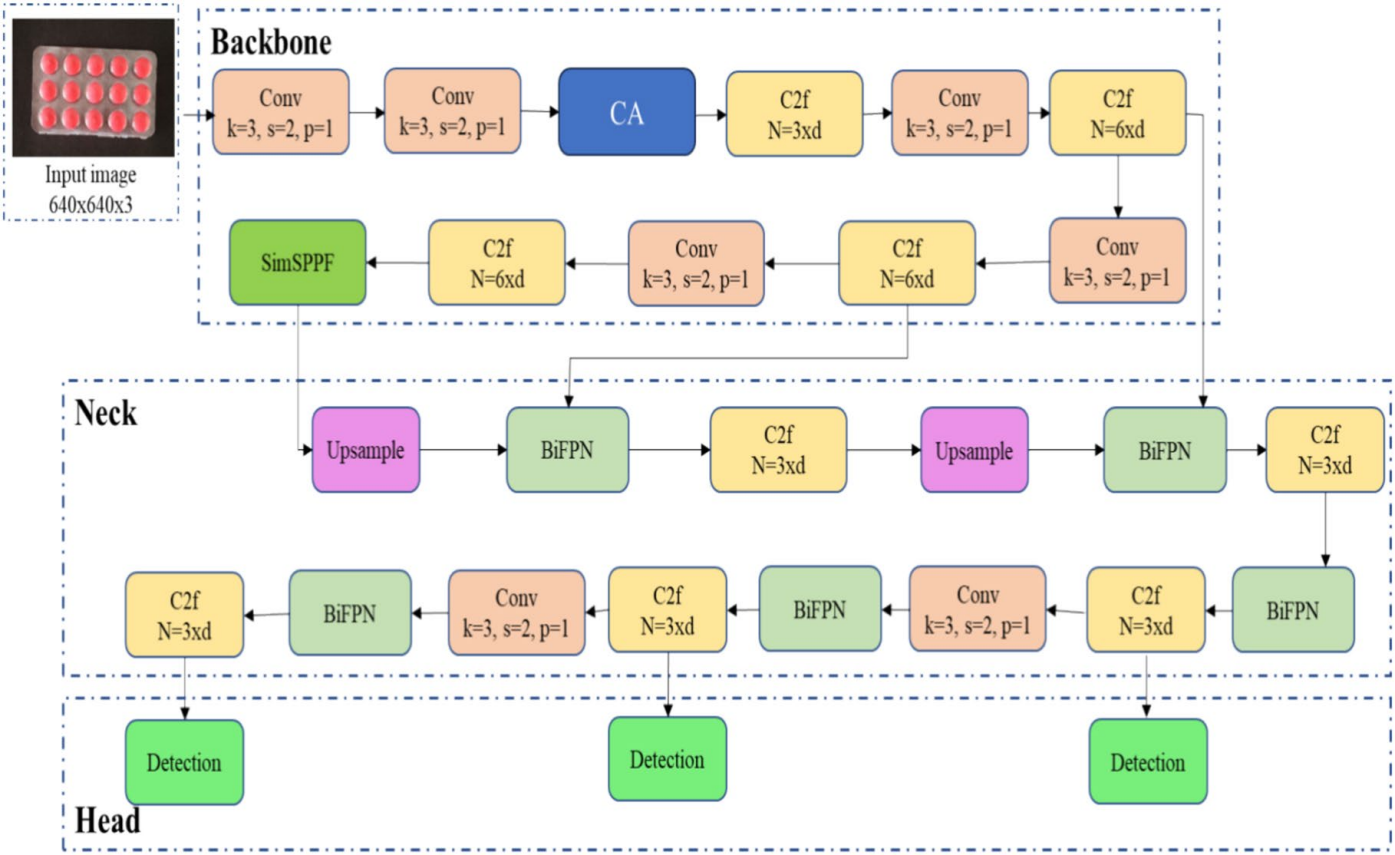
Architecture of CBS-YOLOv8.

Finally, these changes made in YOLOv8 give a more effective and well efficient defect detection model that will work in wide real-time scenarios. The final model gives the tensors of 20 × 20 × 27, 40 × 40 × 27 and 80 × 80 × 27is illustrated in the Fig. 6.

#### Process in blister package defect detection in real-time

**Figure 7:**
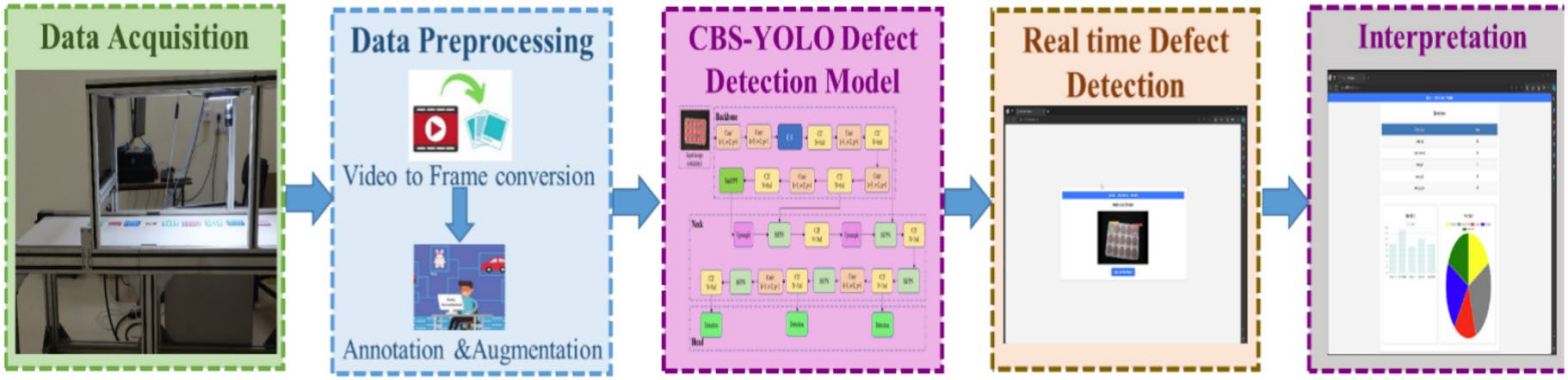
Process involved in CBS-YOLOv8 defect detection model.

The overall process of CBS-YOLOv8 defect detection in real-time environment is shown in Fig. 7. Initially, the video data is acquired through the hardware component and subsequently forwarded to the next stage for preprocessing. Secondly, the video undergoes a conversion into frames, subsequent to which frame-level annotation is executed, followed by an augmentation process, ending in the transformation into the CBS-YOLOv8 detection model. Finally, the proposed model detects the defects both in uploaded video and in real-time video. Then, the results are presented by indicating the number of defects observed within the video.

## Experiment and result analysis

Several experimentations are examined on the custom dataset to prove that the proposed CBS-YOLOv8 model can increase the accurateness and speediness of the object detection. The subsequent sections delineate the dataset particulars, model configuration, performance metrics employed in the proposed model, and the ensuing experimental findings.

### Dataset details

This section describes about the how the dataset for tablet defect detection is created, then how annotation is performed on the defective classes and ends with image augmentation technique.

**Figure 8:**
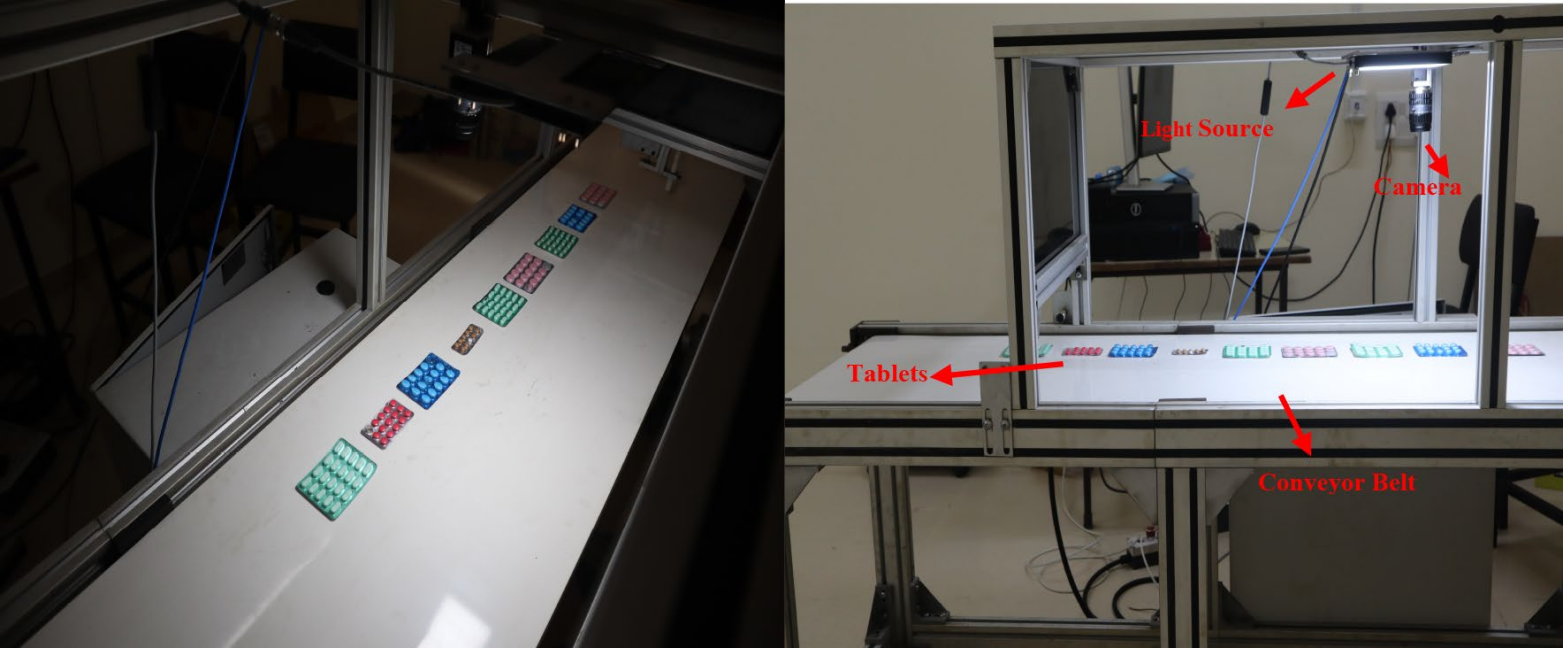
Dataset collection. Blister packages were distributed on the conveyor belt and the video was captured using basler camera under the proper illumination.

**Figure 9:**
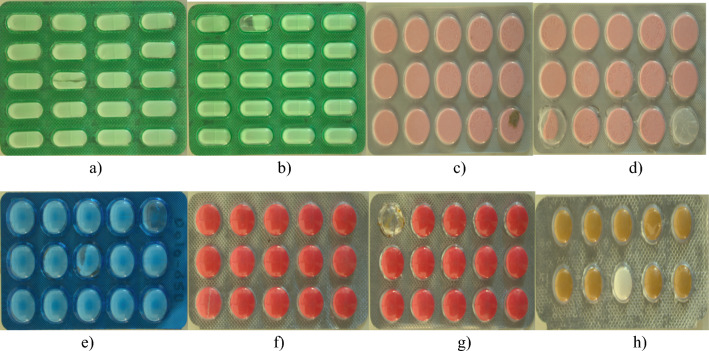
Defective Tablets. (**a**) and (**b**) specify Metformin tablets with defects cracked and broken. (**c**) and (**d**) specify Digene tablet with defects foreign particle, broken and empty. (**e**) specify Dolo-650 tablet with defects empty and broken. (**f**) and (**g**) specify Brufen tablet with defects broken and empty. (**h**) specify Diclofenac tablet with defect color mismatch.

- *Dataset collection* The dataset for both defective and defect free tablets within the blister packages are distributed on the conveyor belt which is operated at the frequency 12 Hz and the videos are captured using Basler camera to attain a real-world detection. Five types of defective classes for five different tablets were created for defect detection in blister packages. The defective tablets include broken tablets, empty tablets, foreign particles, cracked tablets and color mismatches. Tablet types included in the dataset are digene, dolo-650 mg, brufen-200 mg, metformin hydrochloride-500 mg and diclofenac sodium-50 mg. Figure 8 depicts the arrangement for the collection of the dataset. Figure 9 represents the types of defective tablets used in the experiments.
- *Dataset description* The SESOVERA-ST saline bottle fill level monitoring dataset consists of images of saline bottles at different fill levels (empty, 50%, 80%, and 100%). It was created to explore tiny machine learning applications for automating the monitoring of intravenous fluid levels. The dataset includes 4217 original images captured with a Realme X2 mobile phone. These images were augmented and resized to produce a larger dataset of approximately 40,000 images at 64 × 64 pixel resolution. The bottles were photographed from various angles, distances, and backgrounds. The dataset also comes with preprocessing tools for resizing, filtering, and augmentation. It is suitable for tasks such as fill level classification, object detection, and image segmentation. A convolutional neural network tested on this dataset achieved around 94% accuracy. Developed in collaboration between STMicroelectronics and Sesovera.ai, this publicly available dataset aims to enhance efficiency in medical settings and mitigate the risks associated with empty saline bottles^32^.
- *Data annotation* Data annotation plays a crucial role by acting as a bridge between raw data and intelligent algorithms. This process involves exactly adding informative labels or tags to individual data points within a dataset. It empowers machine learning algorithms to grasp the underlying patterns and relationships within the dataset. Automatic label assignment for object detection is also proposed by using center weighting fusion method. Roboflow is an online tool used for the annotation process. In roboflow, the videos are converted into frames, and each frame is annotated if it has defective tablets. Finally, the dataset is annotated for all classes, which includes the total quantity of defects is 3984, and the quantity for each class is mentioned in Table 1. The dataset specifies the quantity of broken tablets is low compared to other defects, and cracked tablet has a high quantity at all. So this leads to a class imbalance problem, which means when the model is trained on this dataset, the prediction is not accurate because the model is trained more on cracked tablets and less on broken tablets.
- *Data augmentation* Data augmentation, utilized in machine learning and deep learning, involves artificially enlarging a training dataset by implementing diverse transformations on existing data samples. These transformations commonly involve actions like rotation, flipping, cropping, scaling, shearing, translation, adjusting brightness and contrast, as well as injecting noise. Data augmentation can help mitigate the class imbalance problem by artificially creating more samples for underrepresented classes, and it can balance the distribution of classes. The increased representation of minority classes can enhance the model's capacity to recognize their distinctive attributes and produce more precise predictions for those specific classes. The data augmentation is done using the roboflow tool, which includes flipping horizontally, performing rotation between − 15° and + 15°, applying grayscale to 54% of images, applying saturation between − 61% and + 61%, brightness between − 25% and + 25% and set the exposure between − 16% and + 16%. After the augmentation process, the dataset is again annotated, yielding a total quantity of 7926 images. This dataset is splits into train dataset, valid dataset and test dataset in the ration 8:1:1. The training dataset comprises 7058 defect images, the validation dataset comprises 650 defective images and the test dataset contains 218 defect images. After splitting the dataset, the distribution of train, valid and test datasets for all defective classes is represented in Table 2. Now, a balanced dataset has been attained, leading to enhanced generalization.

**Table 1:** Quantity of defective classes.

Tablet defect categories	Quantity
Broken tablet	654
Cracked tablet	957
Empty tablet	726
Foreign particle	791
Color mismatch	856

**Table 2:** Distribution of defective classes in train, valid and test dataset.

Tablet defect categories	Training	Validation	Testing
Broken tablet	1427	124	41
Cracked tablet	1404	141	47
Empty tablet	1384	121	42
Foreign particle	1415	126	39
Color mismatch	1428	138	49

### Performance evaluation metrics

- *Precision* Precision evaluates the correctness of positive predictions. It is determined by dividing the count of true positives (TP) by the total of true positives and false positives (TP + FP), where FP denotes false positives.
  11 $$Precision= \frac{TP}{TP+FP},$$
- *Recall* Recall, also referred as a true positive rate (TPR) or sensitivity, indicating the capacity of a model to appropriately recognize actual positive instances. Essentially, it indicates the model's ability to accurately identify all relevant positive cases. Recall is calculated by dividing the number of true positives (TP) by the sum of true positives and false negatives (TP + FN), where FN denotes false negatives.
  12 $$Recall= \frac{TP}{TP+FN},$$
- *F1 score* The F1 score, also known as the balanced F-score or F-measure, is a widely utilized metric for assessing the classification model's performance. It encompasses two key metrics: recall and precision, providing a complete view of the model's accuracy.
  13 $$F1 score=\frac{2\times Precision\times Recall}{Precision+Recall},$$
- *Mean average precision (mAP)* mAP calculation entails computing the average of individual AP (Average Precision) scores for individual category within the dataset. The AP evaluates the model precision and recall for a specific object class.
  14 $$mAP= \frac{1}{N}\sum_{i=0}^{N}{AP}_{i},$$

where N represents the number of classes used in the model. In multi-object classification, the accuracy (mAP) for each category in a model is represented as the average value of AP. mAP@0.5 specifies the mean average correctness of the IoU metric at a threshold of 0.5. mAP@0.5:0.9.5 denotes the mAP mean value for the IoU parameter within the threshold 0.5 to 0.95.

### Model configuration, training and real-time testing

- *Experimental environment setup* The experiments and tests of the tablet defect detection are conducted on the system equipped with GeForce RTX A4000 GPU, Intel(R) Core (TM) i7-12700@2.10 GHz CPU and 64-bit Windows 11 Pro operating system. For model experiments, Python language and PyTorch framework are used, and the model is trained using GPU acceleration all are shown in the Table 3.
- *Model configuration and training* YOLOv8 versions have hyperparameter configuration for training the model, which increases the accuracy. In these experiments, consistent hyperparameters are utilized for all training processes of YOLOv8 is shown in the Table 4.

**Table 3:** Environmental configuration.

Environmental parameters	Value
Operating system	Windows 11 Pro
Deep learning framework	Pytorch 2.2.0 + cu121
GPU	GeForce RTX A4000 GPU
RAM	128 GB
CPU	Intel(R) Core (TM) i7-12700@2.10 GHz
Programming language	Python 3.9

**Table 4:** Configuration of the model.

Hyperparameters	Value
Image size	640 × 640
Optimizer	Auto
Epochs	20
Batch size	AutoBatch
YOLOv8 version	YOLOv8s

**Figure 10:**
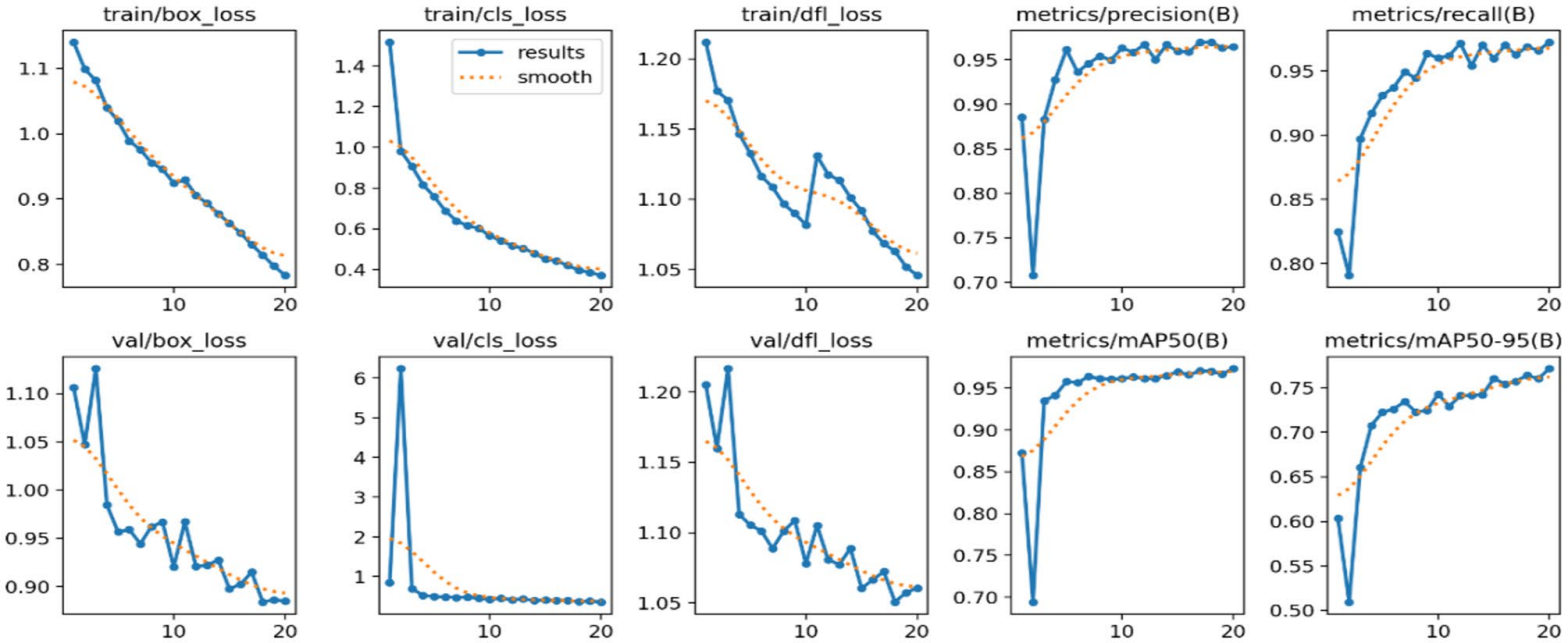
Loss function representation for classification loss, box loss and DFL loss for both training and validation of the CBS-YOLOv8 model on custom dataset.

**Figure 11:**
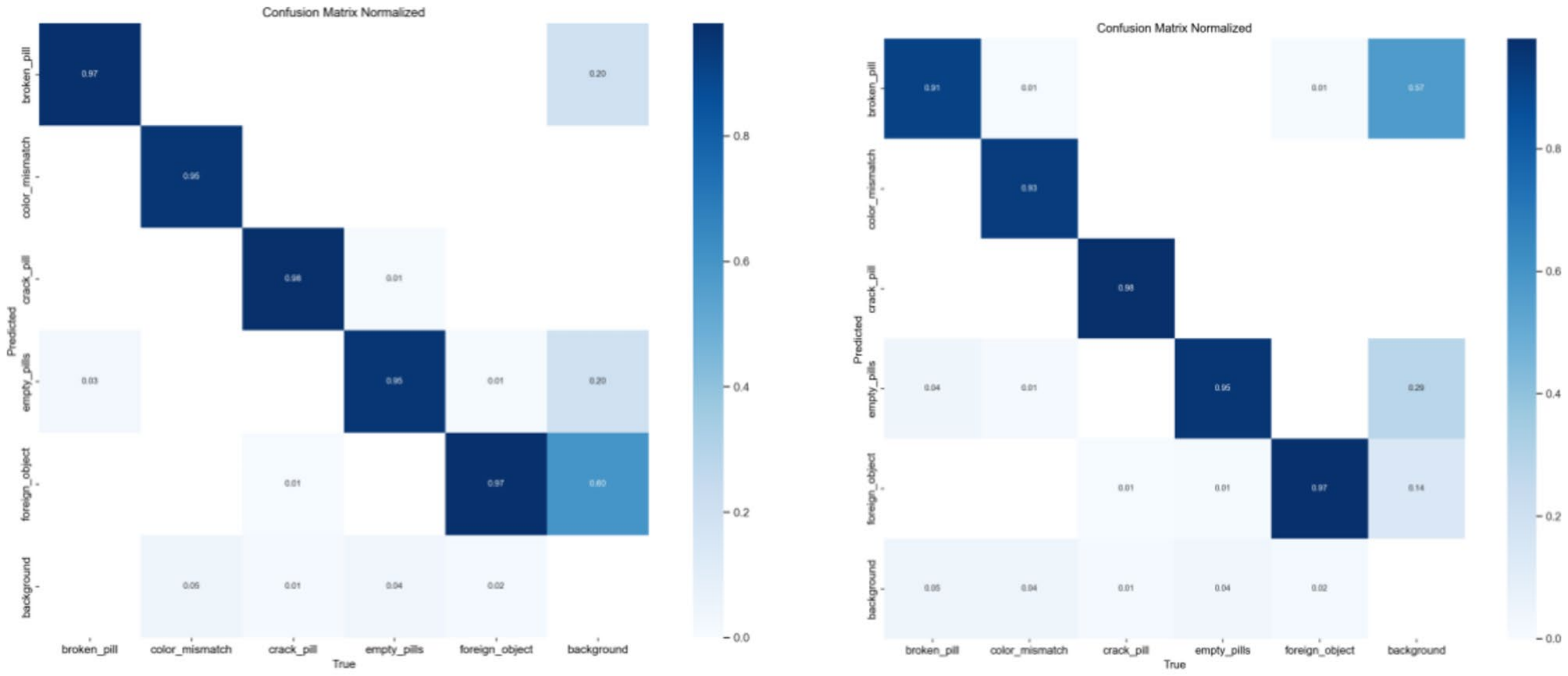
Normalized confusion matrix for CBS-YOLOv8 and YOLOv8s on custom dataset.

**Figure 12:**
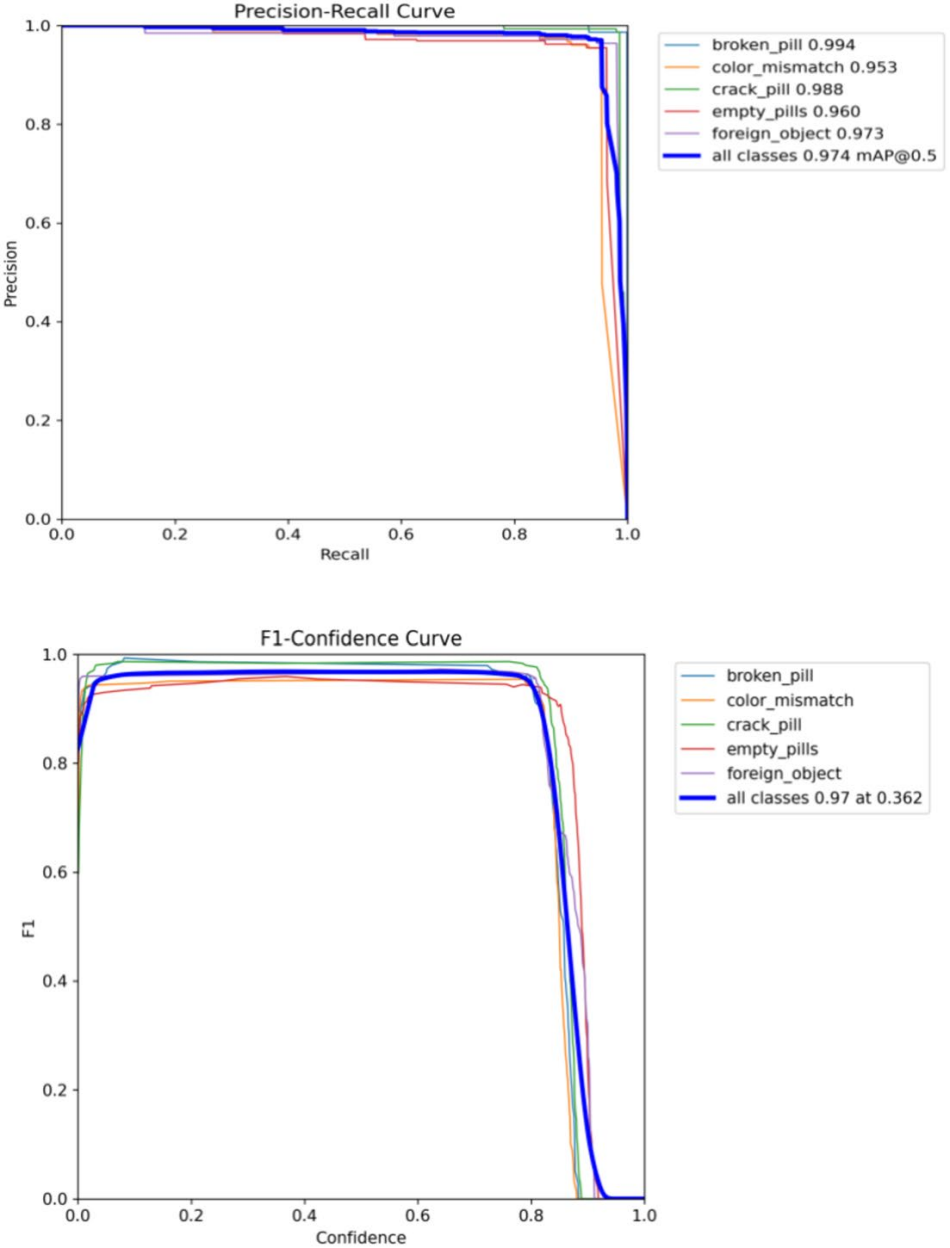
PR (mAP@50 value) and F1-score curve for CBS-YOLOv8 model on custom dataset.

This section gives a complete analysis of the performance of the CBS-YOLOv8 model on custom dataset; training is based on YOLOv8s. Figure 10 represents the loss value of the CBS-YOLOv8 trained dataset, which is plotted against the epoch value. As the epoch rate increases the value for loss will decrease. Once epoch 20 is reached, the mean value for loss function value becomes saturated. Figure 12 shows the obtained result of the F1 score and mAP@0.5 for the CBS-YOLOv8 model, as it shows the highest confidence value in the F1 score and the highest precision and recall curve value, respectively. The proposed CBS-YOLOv8 achieved a mAP of 97.4% and an F1 score of 97%. Then, the CBS-YOLOv8 model was evaluated on the validation dataset, forming a normalized confusion matrix, as shown in Fig. 11. The values along the diagonal within the matrix show the accuracy percentage for each category prediction. Rows indicate predicted categories, columns represent actual categories, and the values found along the crosswise represent the percentage of exact predictions for each category. As compared with YOLOv8s, CBS-YOLOv8 obtained a better confusion matrix (Fig. 12).

**Figure 13:**
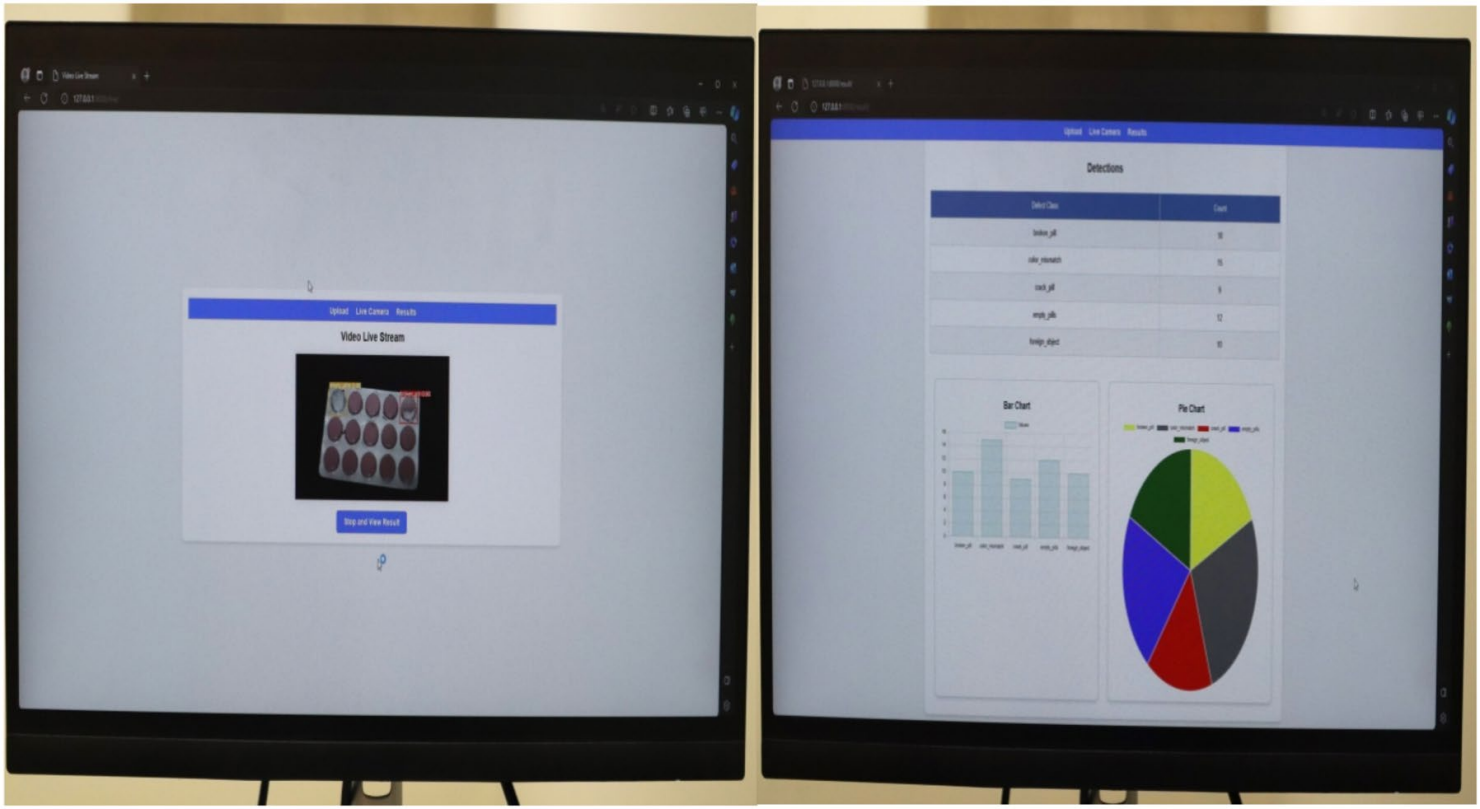
Real-time testing result of CBS-YOLOv8 model.

- *Real-time testing:* After training the dataset, the model is tested using the weight value that is generated while training. The website is created to ensure the real-time defect detection of the pharmaceutical blister packages. The website for defect detection includes the module for both uploaded video testing and live stream video testing. Further, the dashboard is created by showing the defect count for every defect class in the video. Finally, the bar chart and pie chart are visualized, as shown in Fig. 13.

### Ablation study

The investigation aims to assess the improvements behind the CBS-YOLOv8 model and to evaluate how the network's performance is influenced by incorporating a CA, BiPFN, and SimSPPF into the YOLOv8s framework. Following this, the model underwent training and testing on a specially created custom dataset. Table 5 shows the experimental results of a proposed model. The mAP is an important quantitative measure for object detection. In the experiment, mAP is mainly considered as a performance criterion for measuring the accuracy of the proposed CBS-YOLOv8 model. The higher mAP value indicates the model has a higher accuracy.

**Table 5:** Ablation study of CBS-YOLOv8.

Model	All class Precision (%)	All class Recall (%)	mAP50 (%)	mAP50-95 (%)	Para(M)
YOLOv8s	93.6	94.0	95.9	75.5	11.1
YOLOv8s + CA	94.6	95.4	96.5	76.2	48.6
YOLOv8s + CA + BiFPN	95.7	96.5	97.0	76.8	51.8
YOLOv8s + CA + BiFPN + SimSPPF (CBS-YOLOv8)	96.4	97.2	97.4	77.2	35.2

First, the CA module is added to the base network YOLOv8s to increase the extraction of feature capability by increasing the mAP@50 by 0.6% and mAP@50-95 by 0.7%, but the parameter value is increased. Then for achieving the better feature fusion by overcoming the information loss, the BiFPN is added to the above improvement. Now, the model has YOLOv8s, CA module and BiFPN network, which gives the mAP@50 of 97.0% and mAP@50-95 of 76.7% with an increased parameter value of 51.8 M. The mAP@50 and mAP@50-95 are increased by 0.5% and 0.6% respectively. In the above experiment, the parameter value is increased more from the base model; in terms of reducing the parameter value, the SimSPPF is added to improvements, which increase the mAP value and decrease the parameter used in the above experiments. At last, the mAP@50 andmAP@50-95 are increased by 0.4%, and the parameter value is reduced by 16.6 M. Then, the proposed model achieved a mAP@50 and mAP@50-95 for the custom dataset are 97.4% and 77.2%, respectively and from the base model YOLOv8s, the proposed CBS-YOLOv8 model obtained better results with increased mAP@50 by 1.5% and map@50-95 by 1.7%. Precision and recall for all defective classes also increased by 2.8% and 3.2%, respectively.

### Comparison experiment of diverse models

To analyse the superiority and effectiveness of the CBS-YOLOv8 defect detection model on custom dataset, several experiments are conducted to compare the result of CSB-YOLOv8 with diverse models such as two stage detectors including Faster R-CNN^33^ and one stage detector-based methods, including SSD^34^, YOLOv5s, YOLOv7, YOLOv8n, YOLOv8s, YOLOv8m, YOLOv8l, YOLOv8x. The same environmental setup and model configuration are followed for all model comparisons. The comparison of diverse model results with the same dataset is shown in Tables 6 and 7. The detection accuracy of Faster R-CNN, SSD, YOLOv5s, YOLOv7, YOLOv8n, YOLOv8s, YOLOv8m, YOLOv8l, YOLOv8x are 89.3, 86.5, 96.6%, 96.0%, 95.4%, 95.9%, 96.2%, 96.2%, 96.8% respectively. The YOLOv5s achieved high detection accuracy compared to YOLOv8n and YOLOv8s, but the parameter used in YOLOv5s is relatively high to YOLOv8n and in YOLOv7 is high compared to both YOLOv8n and YOLOv8s. So, the YOLOv7 takes more computational time compared to YOLOv5s, YOLOv8n and YOLOv8s. Even though the inference speed of YOLOv7 is less compared to YOLOv5s, which has 50%, and YOLOv8s has a high inference speed of 76.92%. Then the detection accuracy is gradually increased from YOLOv8m to YOLOv8x by 0.6%, but the parameter used in YOLOv8m is 25.8 M, YOLOv8l is 43.6 M and YOLOv8x is 68.1 M leads to very high value. SSD has some missed detection, and Faster R-CNN does not suffer from missed detection but faces multiple bounding box issues. Finally, the CBS-YOLOv8 is proposed for achieving high detection accuracy, high inference speed and low parameter value.

**Table 6:** Comparison results of CBS-YOLOv8 with SSD, Faster R-CNN and various YOLO models on custom dataset.

YOLO versions	mAP (%)					mAP50 (%)	mAP50-95 (%)
	Broken	Color mismatch	Crack	Empty	Foreign particle		
Faster R-CNN	88.6	87.8	86.5	85.7	88.4	89.3	65.8
SSD	86.2	85.2	86.6	84.4	87.1	86.5	62.2
YOLOv5s	99.4	93.5	98.8	94.8	96.6	96.1	75.0
YOLOv7	97.5	93.8	98.3	93.6	96.7	96.0	73.2
YOLOv8n	92.9	94.0	98.8	94.8	96.7	95.4	74.7
YOLOv8s	94.7	94.3	98.6	95.0	96.8	95.9	75.5
YOLOv8m	96.5	93.3	98.7	95.8	96.6	96.2	75.7
YOLOv8l	96.4	93.6	98.6	95.1	97.3	96.2	76.1
YOLOv8x	99.4	93.9	98.7	94.8	97.2	96.8	76.5
CBS-YOLOv8	99.4	95.3	98.8	96.0	97.3	97.4	77.2

**Table 7:** Comparison results of CBS-YOLOv8 with SSD, Faster R-CNN and various YOLO models on custom dataset.

YOLO versions	Para (M)	GFLOP	FPS
Faster R-CNN	28.31	940.97	11
SSD	26.28	62.74	71
YOLOv5s	7.23	15.8	50.0
YOLOv7	37.2	105.2	45.32
YOLOv8n	3.0	8.1	14.28
YOLOv8s	11.1	28.4	76.92
YOLOv8m	25.8	78.7	32.25
YOLOv8l	43.6	164.8	20.83
YOLOv8x	68.1	257.4	16.92
CBS-YOLOv8	35.2	90.1	79.25

In summary, the proposed model has a 97.4% detection accuracy, a high inference speed of 79.25%, and a small decrease in parameter value by 35.2 M. CBS-YOLOv8 has a superior performance compared to YOLOv8s by calculating the detection accuracy, GFLOP, parameter quantity, inference speed, precision and recall.

Tables 8 and 9 present the precision and recall values for all defective classes and compare these metrics across all variations of YOLOv8 alongside the proposed model. When tested on the custom dataset, the YOLOv8n, YOLOv8s, YOLOv8m, YOLOv8l, and YOLOv8x achieve higher precision values are 93.2%, 93.6%, 93.3%, 93.0%, 93.4% respectively, while the CBS-YOLOv8 achieved a precision of 96.4%. For recall, the YOLOv8n, YOLOv8s, YOLOv8m, YOLOv8l, and YOLOv8x achieve better values are 95.3%, 94.0%, 95.4%, 96.0%, and 96.4% respectively, although the CBS-YOLOv8 achieved a recall of 97.2%. Compared with the YOLOv8s, the CBS-YOLOv8’s precision is improved by 2.8%, and recall is increased by 3.2%.

**Table 8:** Precision for all defective class.

YOLO versions	Precision (%)					All class precision (%)
	Broken	Color Mismatch	Crack	Empty	Foreign Particle	
YOLOv8n	82.8	95.3	99.1	92.8	96.2	93.2
YOLOv8s	83.6	95.4	99.3	93.7	96.3	93.6
YOLOv8m	81.5	95.3	99.3	94.6	96.0	93.3
YOLOv8l	84.0	95.1	98.3	93.0	94.7	93.0
YOLOv8x	85.3	95.0	98.4	93.4	95.2	93.4
CBS-YOLOv8	98.6	94.9	98.7	95.4	94.6	96.4

**Table 9:** Recall for all defective class.

YOLO versions	Recall (%)					All class recall (%)
	Broken	Color mismatch	Crack	Empty	Foreign particle	
YOLOv8n	90.2	93.3	98.7	96.4	98.2	95.3
YOLOv8s	86.3	93.3	97.6	94.7	98.2	94.0
YOLOv8m	95.1	91.5	98.1	95.0	97.2	95.4
YOLOv8l	95.1	93.3	98.0	95.5	98.2	96.0
YOLOv8x	95.9	93.8	97.8	95.8	98.0	96.4
CBS-YOLOv8	98.2	95.4	98.0	96.4	98.2	97.2

### Analysis of detection result

**Figure 14:**
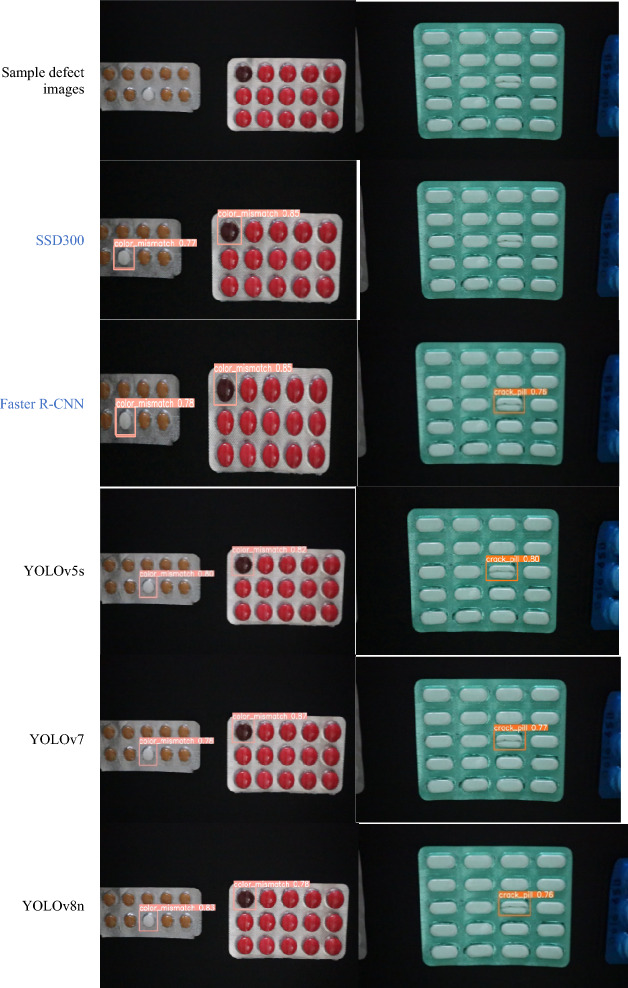

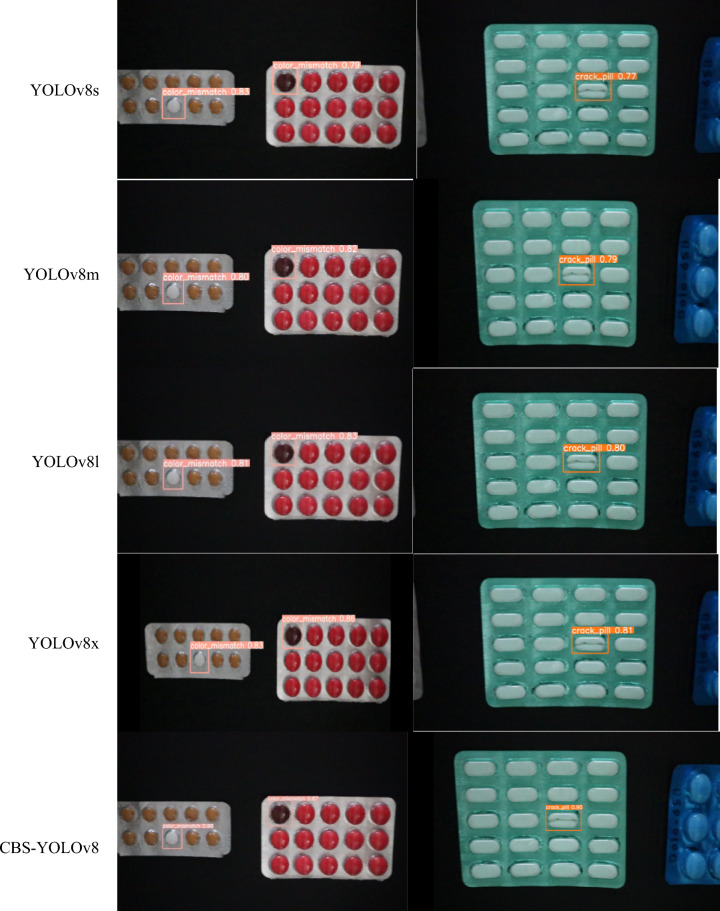
Confidence level comparison for a diverse model with the CBS-YOLOv8 model on custom dataset.

This section examines the test outcomes of all models by displaying their confidence levels, as shown in Fig. 14. The proposed CBS-YOLOv8 gives a better prediction on real-time video when compared to all other models. The comparison for training curves is shown in Fig. 18.

**Figure 15:**
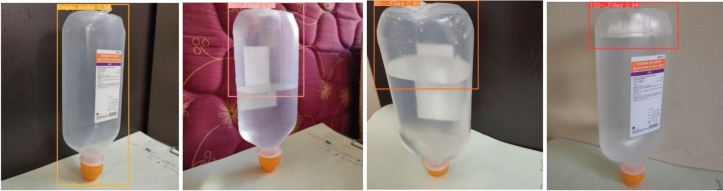
Detection result of CBS-YOLOv8 model on SESOVERA-ST saline bottle fill level monitoring dataset.

**Figure 16:**
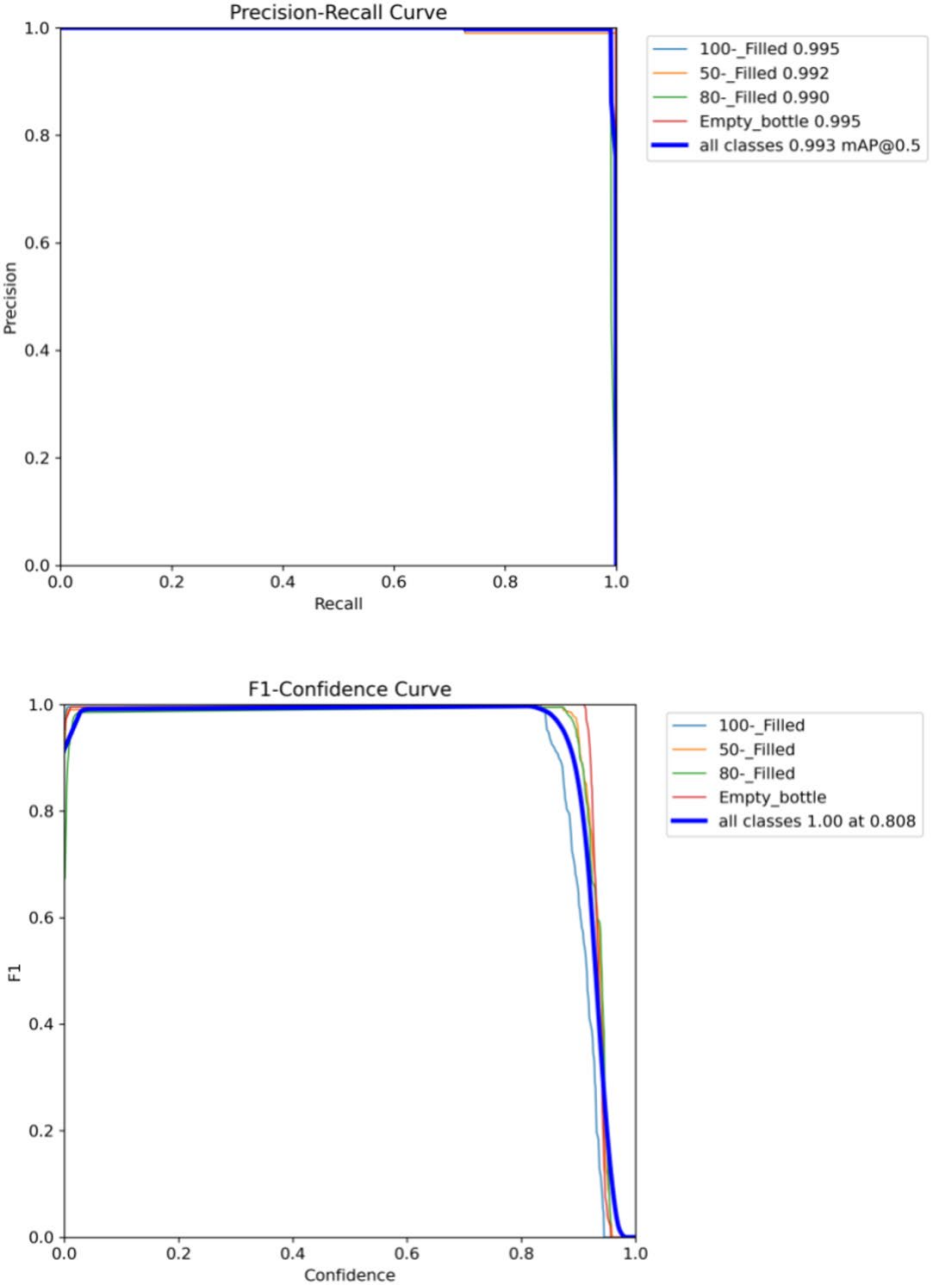
PR (mAP@50 value) and F1-score curve for CBS-YOLOv8 model SESOVERA-ST saline bottle fill level monitoring dataset.

**Figure 17:**
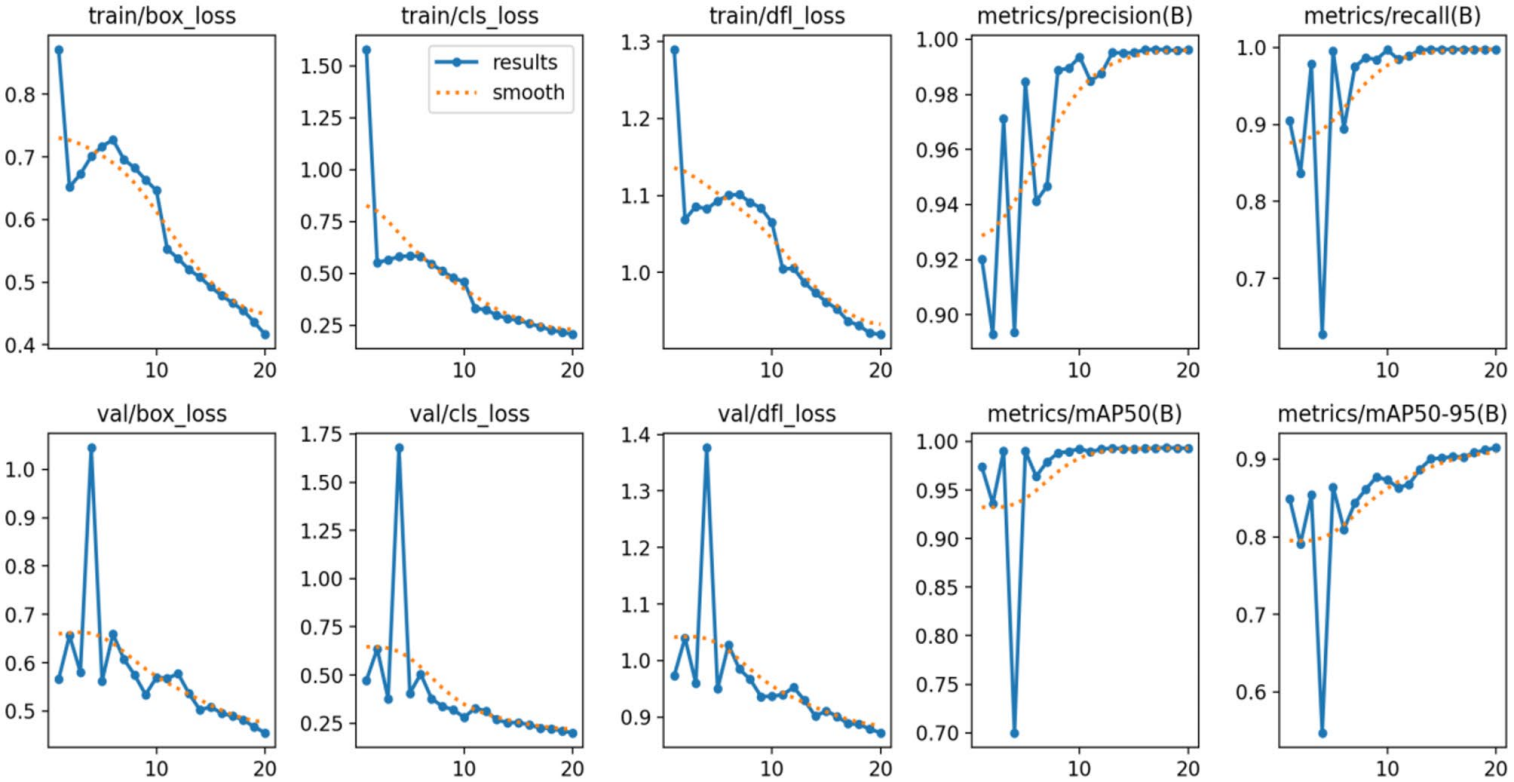
Loss function representation for classification loss, box loss and DFL loss for both training and validation of the CBS-YOLOv8 model on SESOVERA-ST saline bottle fill level monitoring dataset.

The proposed model is also trained on the SESOVERA-ST saline bottle fill level monitoring dataset and gives better results than the other SOTA model, and its detection results with confidence level on the tested images is shown in Fig. 15. The proposed model is achieved mAP50 of 99.3%. Figure 16 shows the obtained result of the F1 score and mAP@0.5 for the CBS-YOLOv8 model, as it shows the highest confidence value in the F1 score and the highest precision and recall curve value, respectively. Figure 17 represents the loss value of the CBS-YOLOv8 trained dataset, which is plotted against the epoch value.

**Figure 18:**
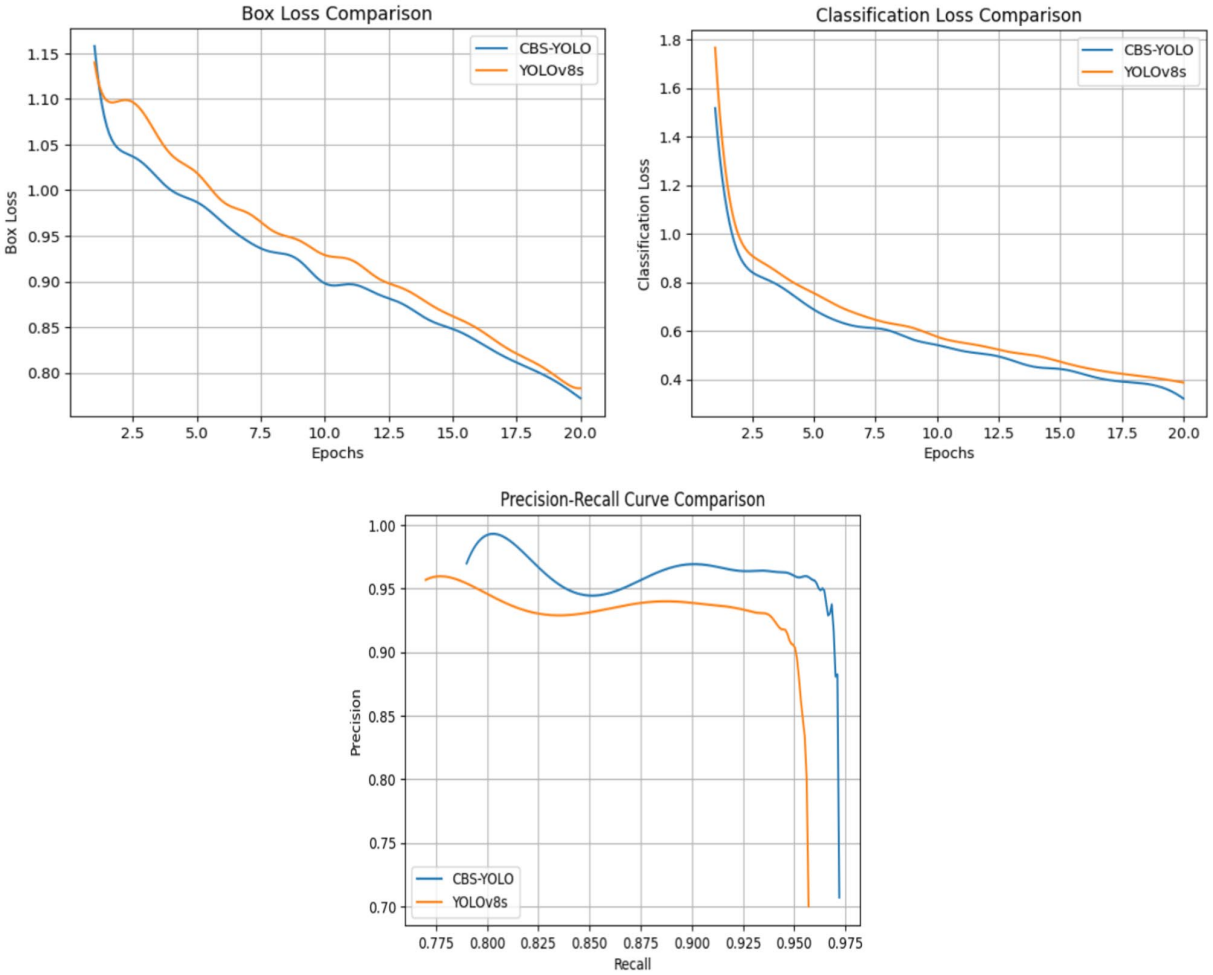
Training box loss curve, training classification loss curve and PR -curve between the YOLOv8s and CBS-YOLOv8 on custom dataset.

The assessment of detection results of Faster R-CNN, SSD, YOLOv5, YOLOv7, all YOLOv8 versions, and CBS-YOLOv8 models are trained on the custom dataset and estimate its performance on the test dataset and real-time video. For each video, the detection result is shown, and its corresponding visualization graph with defect counts is also displayed. In Fig. 14, the CBS-YOLOv8 model achieves the highest confidence value of the other models. As shown in the result, the model correctly identifies the defective tablet with high confidence. Furthermore, the analysis was made on the mAP curve, classification loss, and box loss between the YOLOv8s and the CBS-YOLOv8 model. The CBS-YOLOv8 model has the highest mAP value for all defective classes compared to YOLOv8s. Then, the box loss and classification loss curve for CBS-YOLOv8 is generated, which has a decrease in training loss when compared to the baseline model YOLOv8s. The model is also experimented on the SESOVERA-ST saline bottle fill level monitoring dataset, which gives the efficient detection on the saline bottle for measuring the level of the bottle. These detection results demonstrate that the proposed model attains a high accuracy and inference speed by increasing the YOLOv8s accuracy and inference speed for defect detection in pharmaceutical packaging (Fig. 18).

## Conclusion

In order to increase the feature extraction capacity in YOLOv8, this research work proposes the CBS-YOLOv8 model for detecting defects in pharmaceutical blister packages. This model incorporates the CA mechanism in the backbone of the YOLOv8 model to increase the number of target features. Additionally, includes the BiFPN module in YOLOv8’s neck part to enhance the multi-scale feature fusion process to minimize the important information loss while doing upsampling and downsampling because the images are in different scales. At last, to increase the inference speed of the proposed CBS-YOLOv8 model, the SPPF presented in the backbone of YOLOv8 is changed with SimSPPF, which reduces the computational overhead. The experiments are done on the custom dataset of tablet defect detection and compared with other versions of the YOLO family. The experimental results describe that the proposed CBS-YOLOv8 attains a notably higher performance than the other models. When equated with the baseline model YOLOv8s, the proposed model achieves a mAP50 of 97.4% on custom dataset and an inference speed of 79.25FPS, which are increased by 1.5% and 2.3%, respectively. The proposed model is also tested on the SESOVERA-ST saline bottle fill level monitoring dataset which gives mAP50 of 99.3% with high confidence value. However, the CBS-YOLOv8 achieves a better trade-off in terms of accuracy and speed. In future research, the integration of YOLO with Human-in-the-Loop (HITL) systems will be explored for managing manufacturing processes during unforeseen scenarios in which AI lacks programming to address critical situations.

## Data Availability

The datasets produced and/or analysed in this study can be obtained from the corresponding author upon request, subject to reasonable terms.
